# Supplementary material for: Magic number colloidal clusters as minimum free energy structures
Source: Nat Commun. 2018 Dec 10;9:5259. doi: 10.1038/s41467-018-07600-4 (PMC6288123; doi:10.1038/s41467-018-07600-4)
Supplement: Supplementary file 1 — Supplementary Information [file 41467_2018_7600_MOESM1_ESM.pdf]

Supplementary Information for

Magic Number Colloidal Clusters as Minimum Free Energy Structures

Junwei Wang[1], Chrameh Fru Mbah[2], Thomas Przybilla[3], Benjamin Apeleo Zubiri[3], Erdmann Spiecker[3], Michael Engel[2,*], Nicolas Vogel[1,*]

[1]Institute of Particle Technology, [2]Institute for Multiscale Simulation, [3]Institute of Micro- and Nanostructure Research, Friedrich-Alexander University Erlangen-Nürnberg, 91058 Erlangen, Germany.

*Corresponding authors: michael.engel@fau.de, nicolas.vogel@fau.de

Contents of Supplementary Materials:

1 Supplementary Figures 2

1.1 Fabrication of Colloid Clusters 2

1.2 Geometric Model of Magic Number Colloidal Clusters (MCCs) 5

1.3 Electron Tomographic Confirmation of the Model 6

1.4 Library of MCCs 8

1.5 Formation and Kinetics of MCCs from Simulation 10

1.6 Thermodynamic Stability of MCCs 14

1 Supplementary Figures

1.1 Fabrication of Colloid Clusters

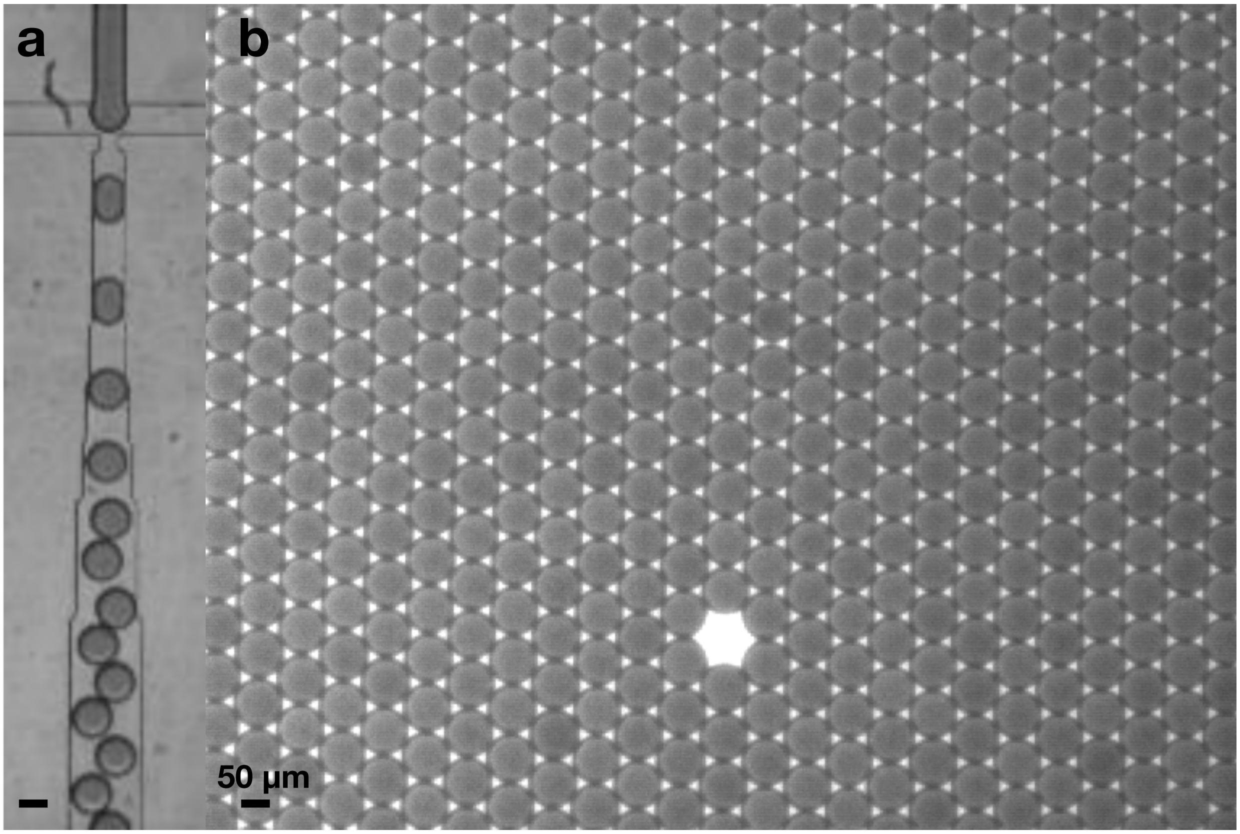

Supplementary Figure 1 | Monodispersed droplet fabrication by microfluidic device. a, Water in oil droplet formation in cross-junction PDMS microfluidics. 244 nm PS colloidal particles are dispersed in the water phase. **b**, Large quantity of monodisperse droplets, stabilized by PFPE-PEG surfactant.

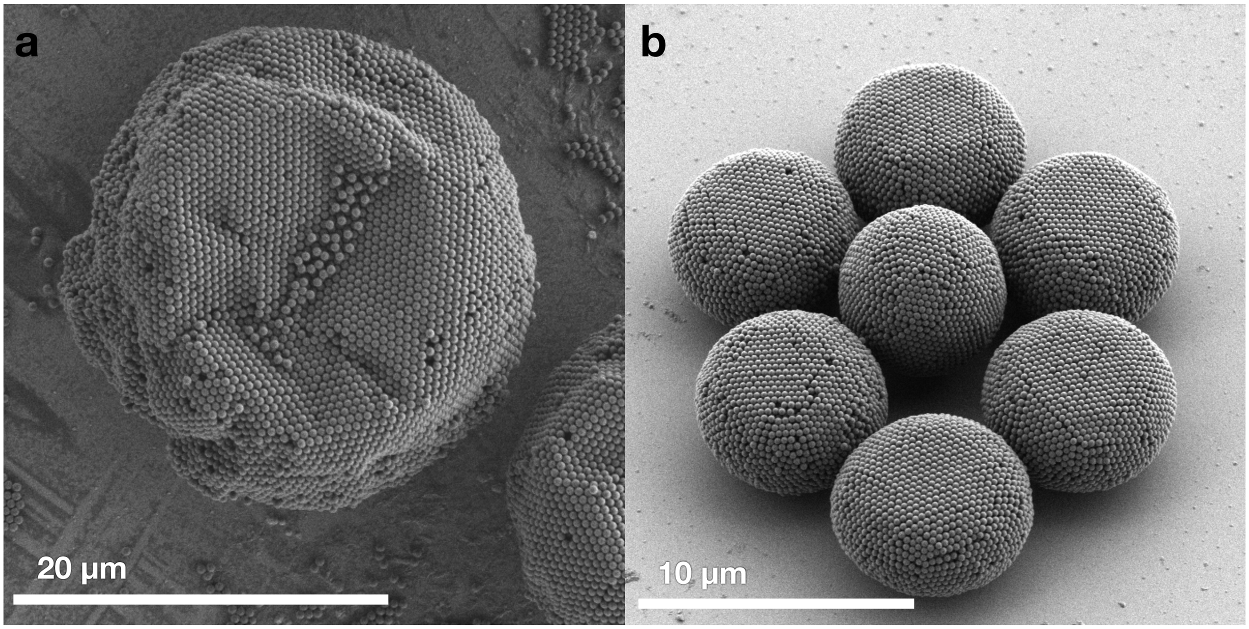

Supplementary Figure 2 | Buckled colloidal cluster obtained with fast water evaporation rate. **a**, Scanning electron microscope (SEM) image of a heavily buckled colloidal cluster with morphology of a deflated football, caused by rapid water evaporation before consolidation of colloids in the emulsion droplet. **b**, Six weakly buckled colloidal clusters surrounding a spherical cluster, observed at around 30-degree viewing angle. The buckled clusters, containing a similar number of colloids as the spherical cluster, appear flatter and larger in diameter.

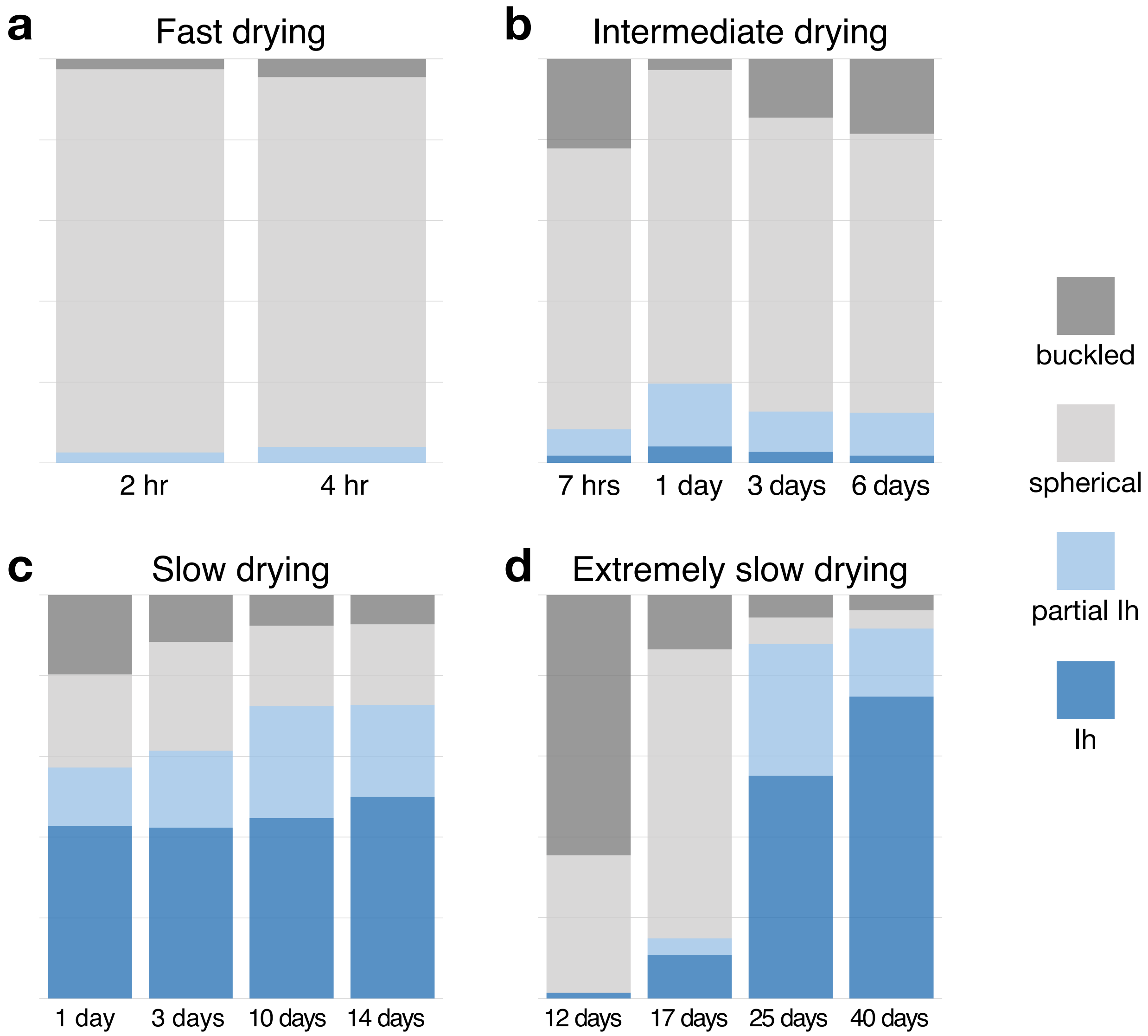

Supplementary Figure 3 | Influence of water evaporation rate and evolution of cluster type over time. Samples are kept in 85 °C (**a**), 25 °C (**b**) and 5 °C (**c, d**) with different water evaporation rate. Samples in (**c**) are kept in a container vial with a larger opening for faster water evaporation rate than samples in (**d**). Results in (**c**) and (**d**) demonstrate that water evaporation rate, instead of temperature, is responsible for the observed difference in cluster types. Slower water evaporation rate leads to more icosahedral clusters. Icosahedral clusters of the investigated size form in a single droplet in the time scale of a few days.

1.2 Geometric Model of Magic Number Colloidal Clusters (MCCs)

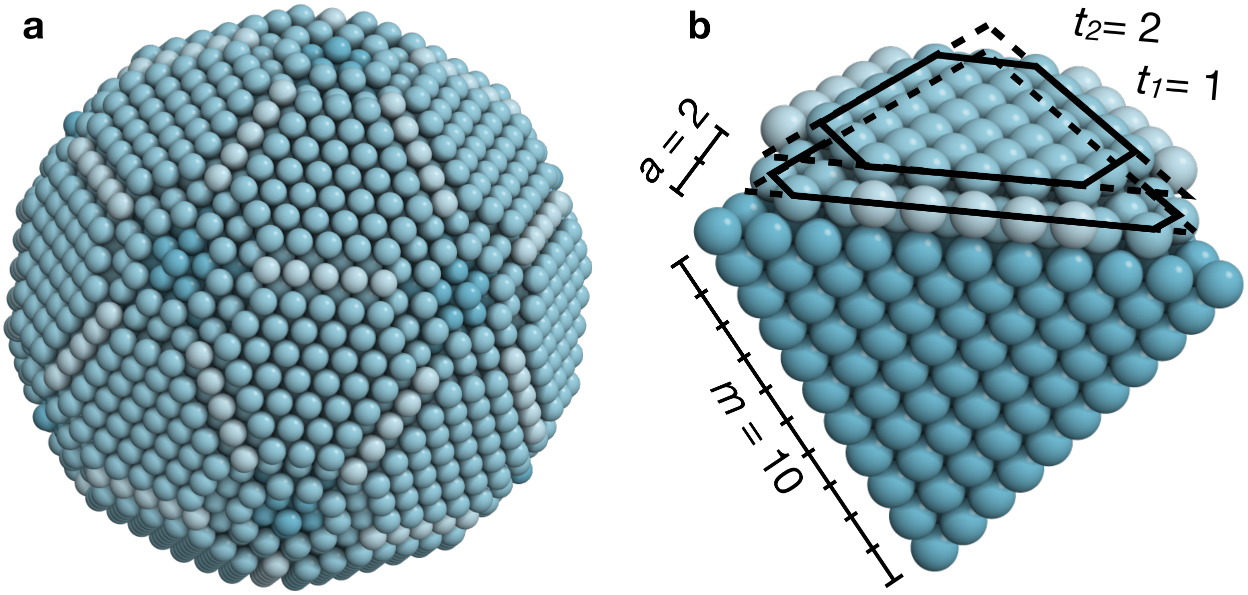

Supplementary Figure 4 | Modelling and notation of MCCs. a, The spherically truncated model accurately reproduces MCC structures observed in experiments. **b**, Close up of one of the twenty tetrahedral building blocks to highlight the Mackay and anti-Mackay shells. This MCC has ten Mackay icosahedral shells, m and two anti-Mackay shells, a. Denoted by the number of total shells and the number of anti-Mackay shells in the subscript, $(m{+}a)_a$, this is a type 12_2 MCC. Determination of detailed surface pattern requires a third truncation parameter t in each shell. In this 12_2 MCC, the triangle in the first anti-Mackay shell is truncated by 1, the second anti-Mackay shell by 2, therefore $t_1 = 1$, $t_2 = 2$. The truncation in each anti-Mackay shell follows $t_a \geq \ldots \geq t_1$. The surface is tiled by rectangles of width $a + 1$ and length $m + 1 - a - 2t_a$ and (truncated) triangles of unshared side $t_a + 1$, in this case 3, 5 and 3. The numbers of particles in the Mackay icosahedron shell is given by Mackay (*6*),

$$\{10 \times (m + 1)^3 - 15 \times (m + 1)^2 + 11 \times (m + 1) - 3\} / 3,$$

the number of particles in each anti-Mackay shells follows

$$10 \times (m + 2 - a_a)\,(m + 1 - a_a) + 30 \times (m + 1 - a_a)\,(a_a - 1) - \{30 \times t_a\,(1 + t_a) + 60 \times t_a\,(a_a - 1)\},$$

where a_a is the sequence number of anti-Mackay shells and t_a is the truncation at this shell. In the present case, for the first anti-Mackay shell, $a_1 = 1$, $t_1 = 1$, for the second anti-Mackay shell, $a_2 = 2$, $t_2 = 2$. Therefore, there are 3871, 1040, and 870 spheres in the Mackay icosahedron core, first anti-Mackay and second anti-Mackay shell respectively, resulting in a total of 5781 spheres in this 12_2 MCC.

1.3 Electron Tomographic Confirmation of the Model

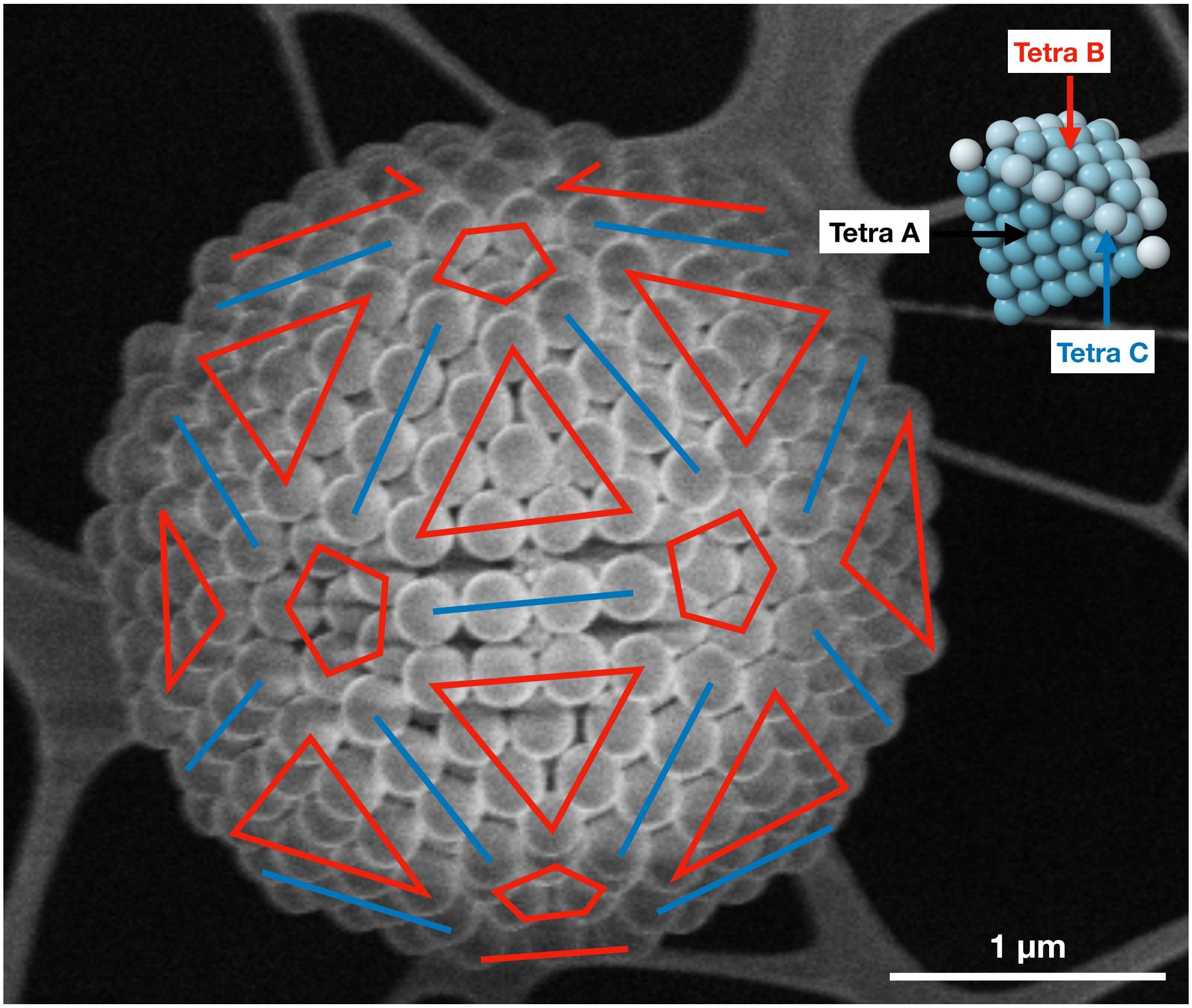

Supplementary Figure 5 | Surface features of type 7_2 MCC are in perfect agreement with model structure. In the SEM image, surface particles, marked by red triangles, are part of the tetrahedra over the faces of the MI core in the model. Particles, marked by blue line segments, are part of the tetrahedra over the edge of the MI core in the model. These particles form the second anti-Mackay shell in the 7_2 MCC. In accordance with the equation described in Supplementary Figure 4, this 7_2 MCC surface is tiled by rectangles of width 3 and length 4, and triangles of unshared side length 1.

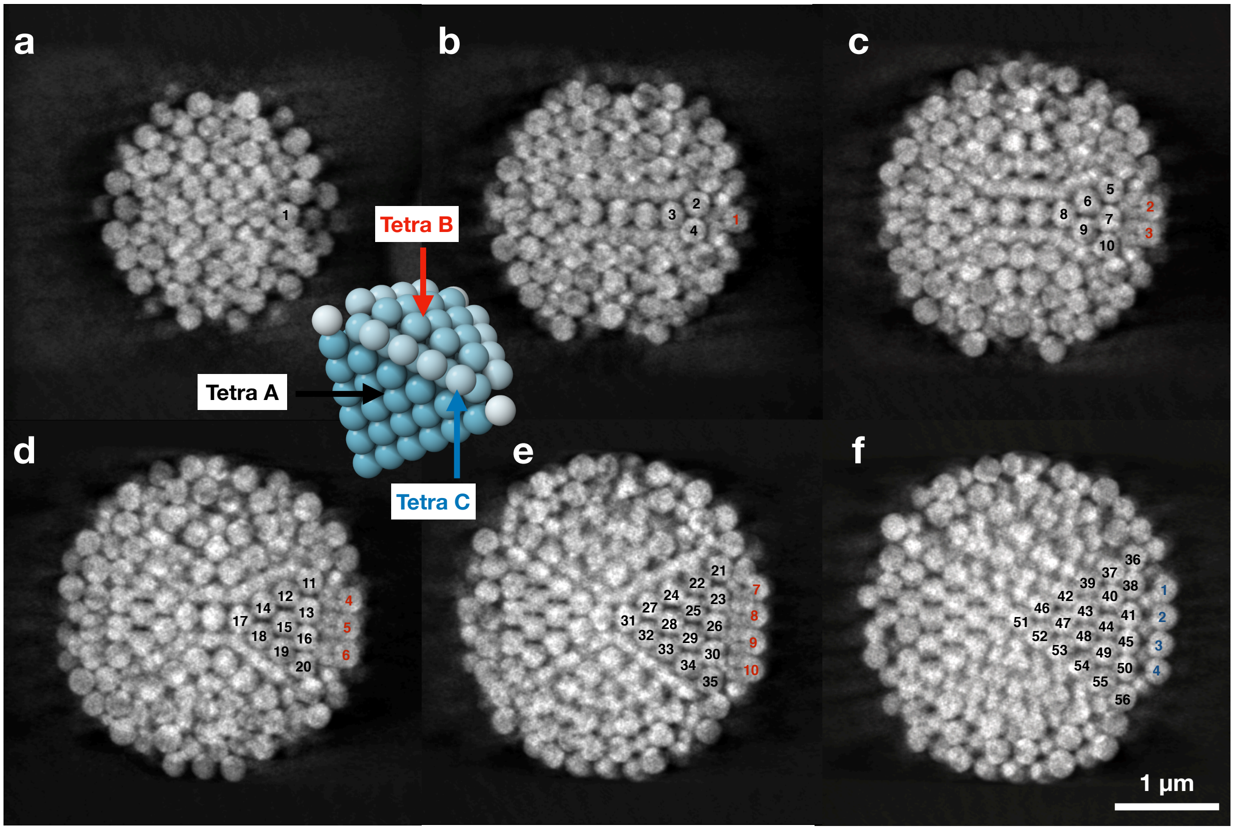

Supplementary Figure 6 | STEM tomography confirms the MCC model. The virtual slices through the three-dimensional reconstruction perpendicular to the cluster's two-fold symmetry axis reveal a highly ordered interior structure at the constituent particle level. The particles marked with black numbers correspond to the five-shell Mackay icosahedron (MI) core, where one tetrahedron grain consists of 56 spheres. The ten particles in red originate from the tetrahedra over the MI faces in the second anti-Mackay shell that form triangles at the cluster surface. The four particles in blue in (**f**) originate from tetrahedra over the MI edges. A careful analysis of the reconstructed cluster identifies 602 out of 643 (94% accuracy) particles at their predicted position by the model in the upper hemisphere.

1.4 Library of MCCs

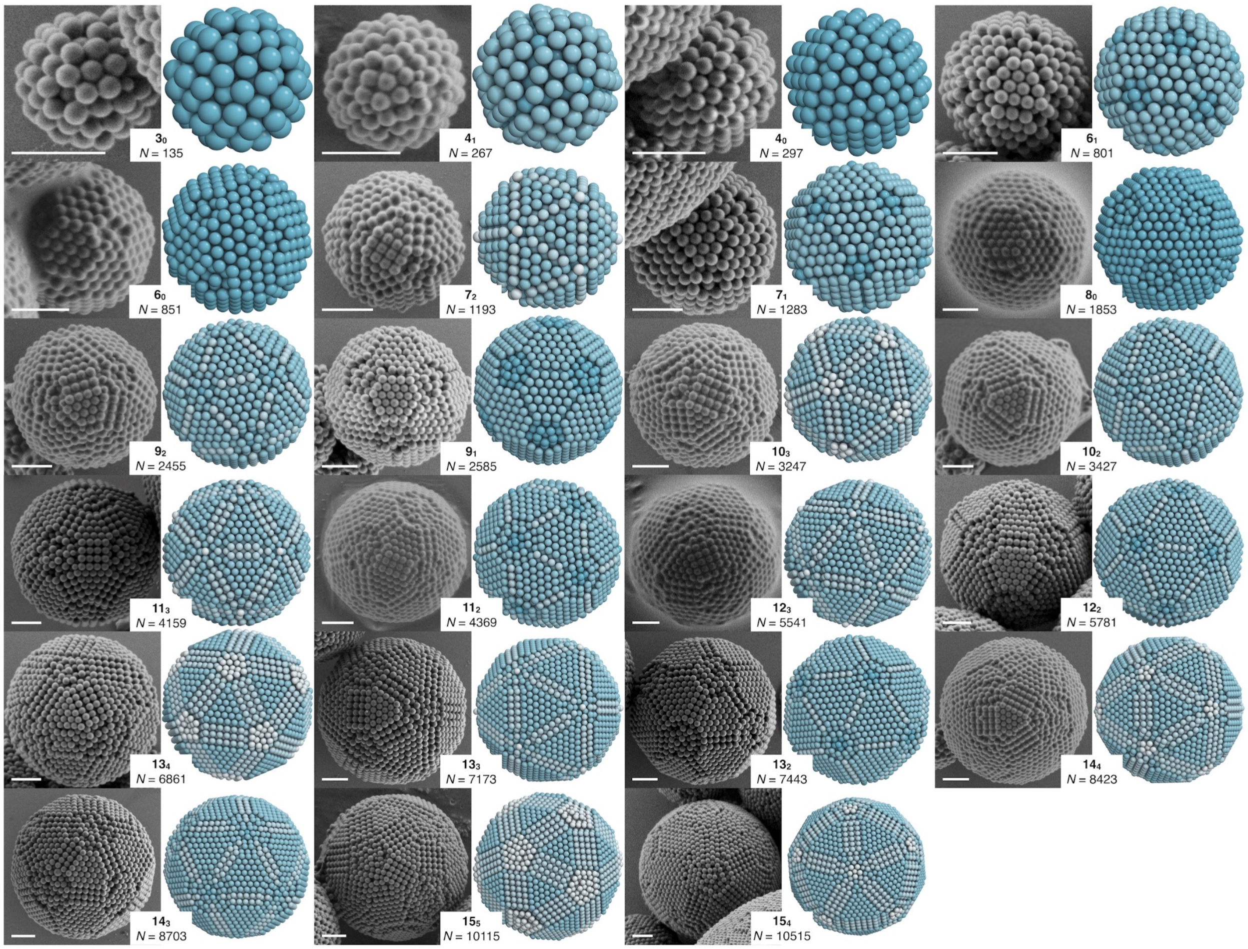

Supplementary Figure 7 | Library of observed MCCs in experiments of sizes ranging from 100 to about 10,000 colloids. All observed MCCs, arranged by increasing system size, are accurately described by our model and denoted by the number of total shells $m + a$ and number of anti-Mackay shells a in subscript. The number of particles in the system N cannot be measured in experiment but is instead taken from the model. MCCs with number of total shells from 3 to 15 were discovered. It is evident that larger numbers of shells can adapt more anti-Mackay shells, but not more than half the number of Mackay shells, $a < m / 2$. Given the same number of total shells, MCCs with more anti-Mackay shells have fewer particles, indicating that adopting anti-Mackay shells is an efficient adaptation to the spherical confining boundary. Scale bars, 1 μm.

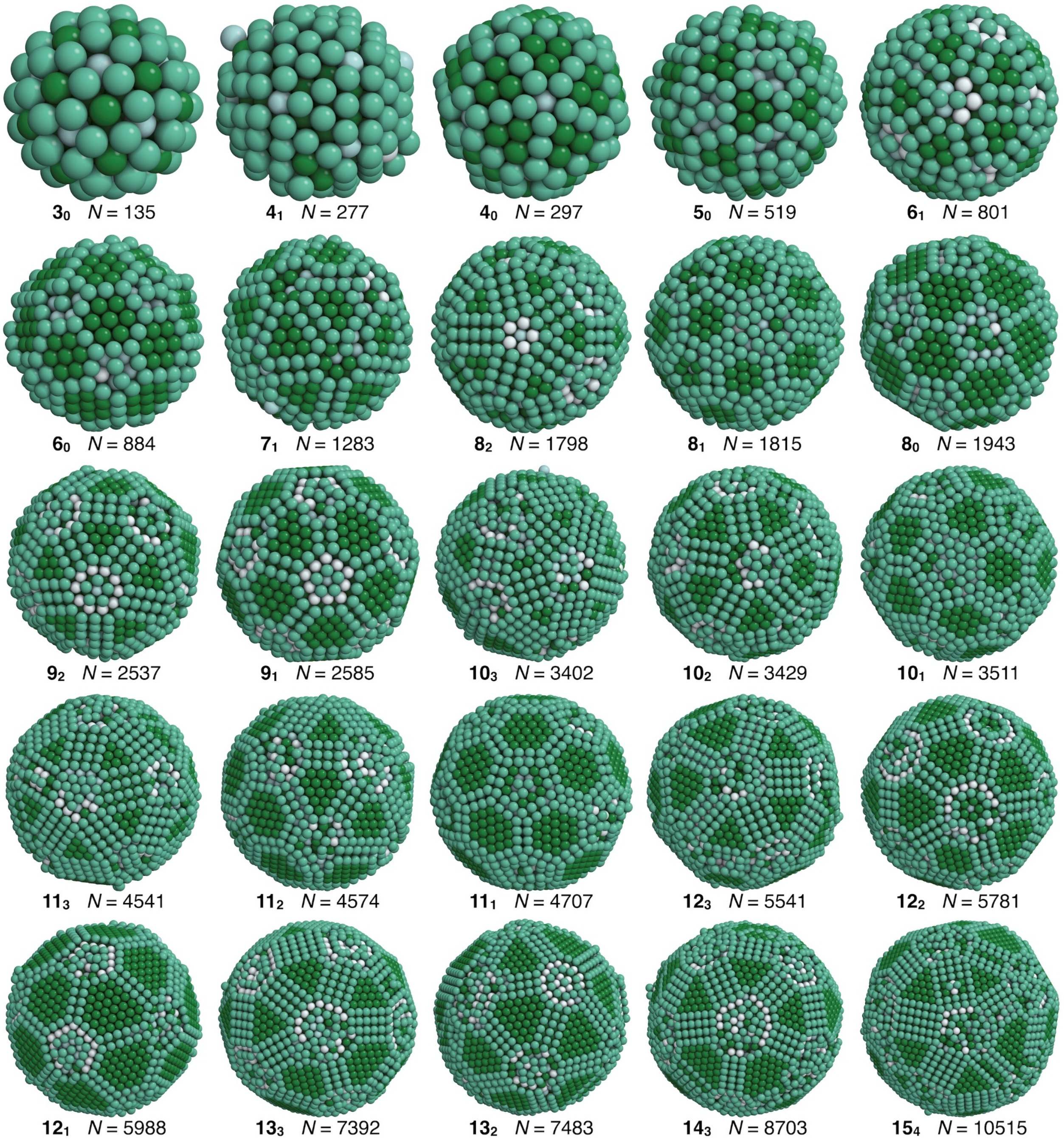

Supplementary Figure 8 | Library of observed MCCs in simulations of sizes ranging from 100 to about 10,000 particles. All observed MCCs, arranged by increasing system size, are accurately described by our model and denoted by the number of total shells $m + a$ and number of anti-Mackay shells a in subscript. The number of particles in the system N is known from the simulation. MCCs with number of total shells from 3 to 15 were discovered. The simulation data compares well to experimental data (Supplementary Figure 7).

1.5 Formation and Kinetics of MCCs from Simulation

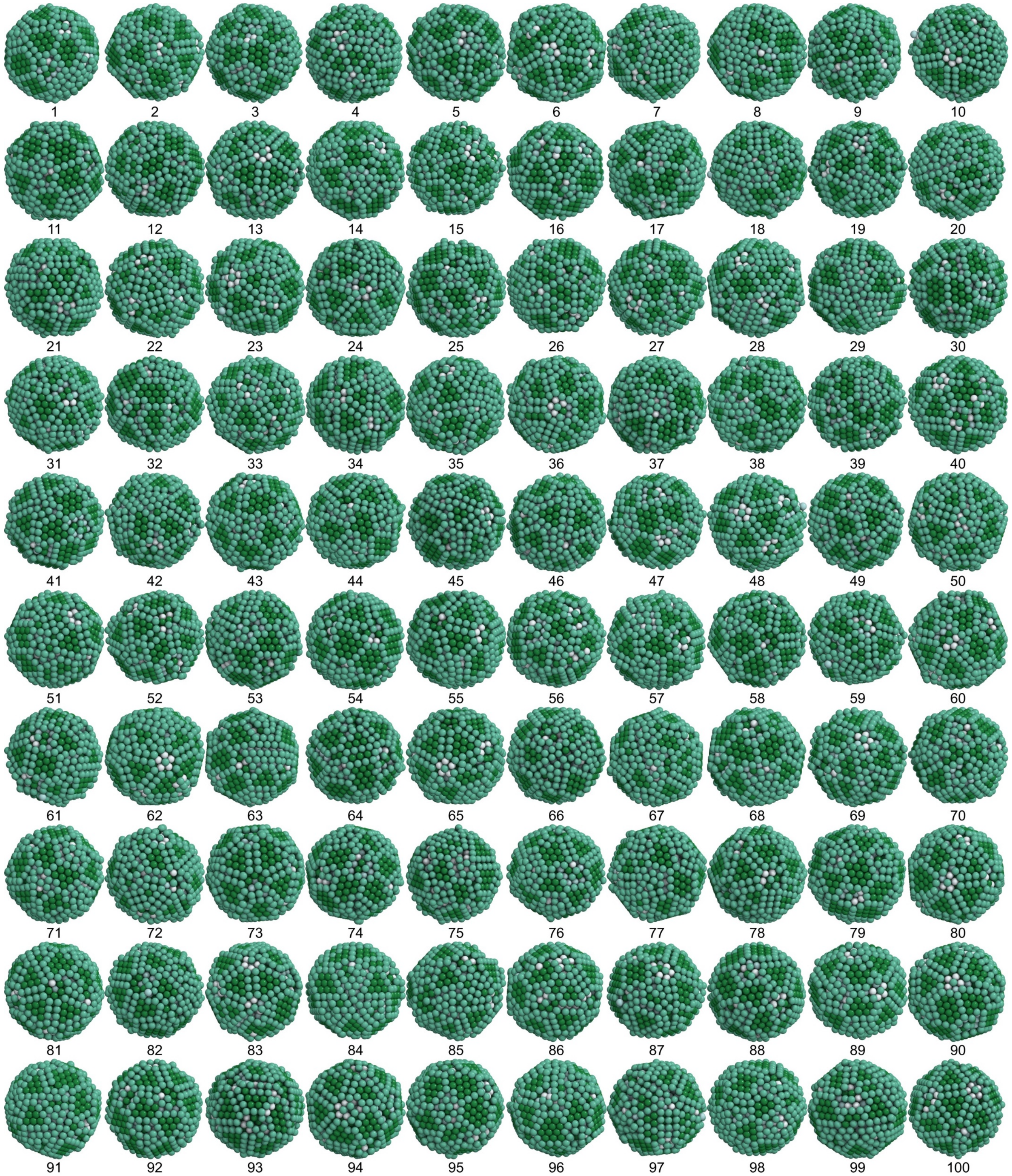

Supplementary Figure 9 | Quenched snapshots of clusters from the 100 equations of state in Fig. 5a at $\phi = 0.55$, $N = 1308$ ($m + a = 7$ shells). All clusters have icosahedral order in the bond orientational order diagrams and a perfect MI core (not shown). The outer shells exhibit characteristic surface features (rectangles and truncated triangles) but also frequently include disorder. We conclude that self-assembly generally leads to icosahedral order but not always to high-quality outer shells for this system size with our quenching protocol.

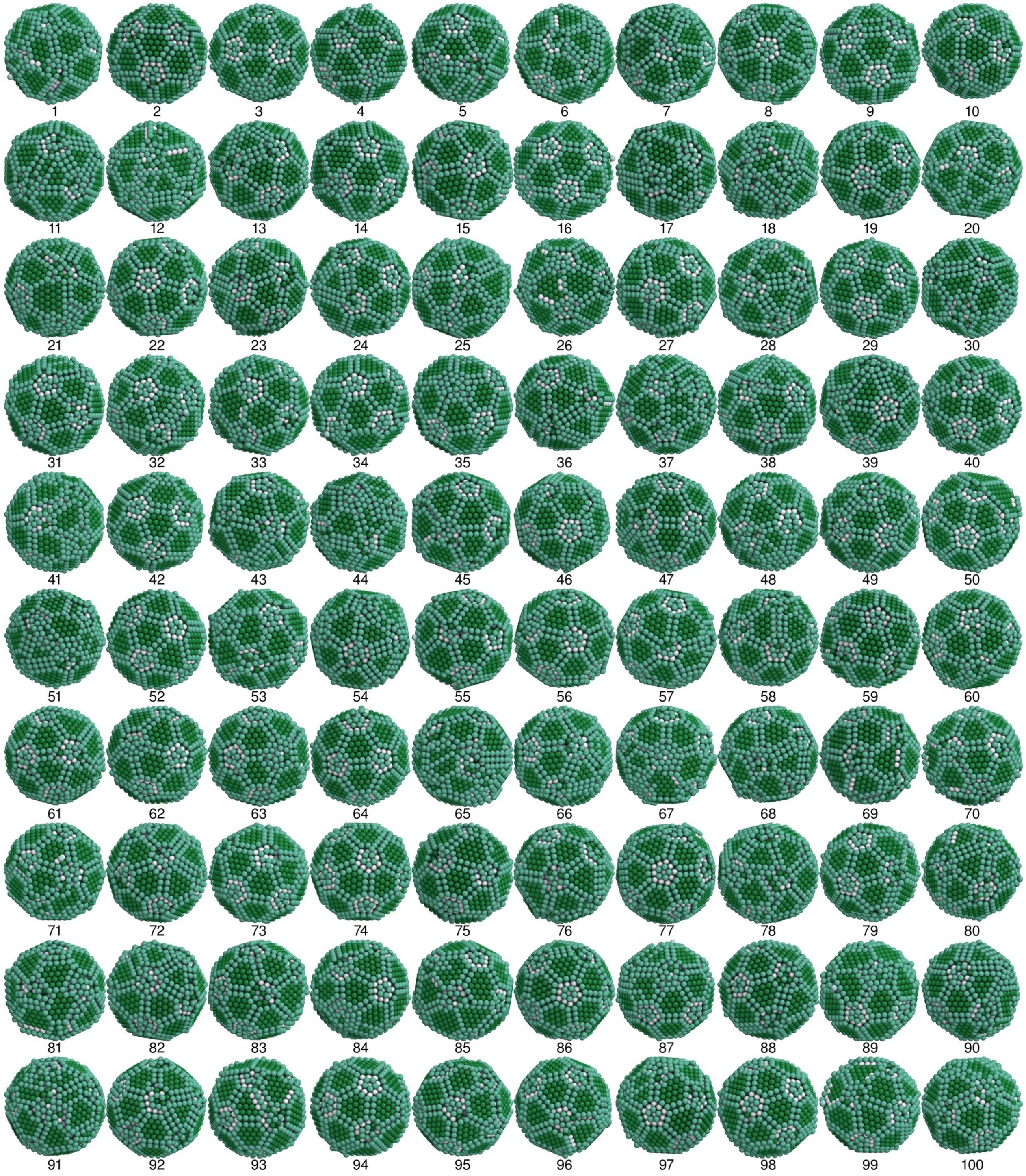

Supplementary Figure 10 | Quenched snapshots of clusters from the 100 equations of state in Fig. 5a at $\phi = 0.55$, $N = 2641$ ($m + a = 9$ shells). All clusters have well-formed characteristic surface features (rectangles and truncated triangles) and are well described as MCCs in the outer shell. A few clusters are without any defects, demonstrating that confined self-assembly into MCCs is highly robust and reproducible for this system size.

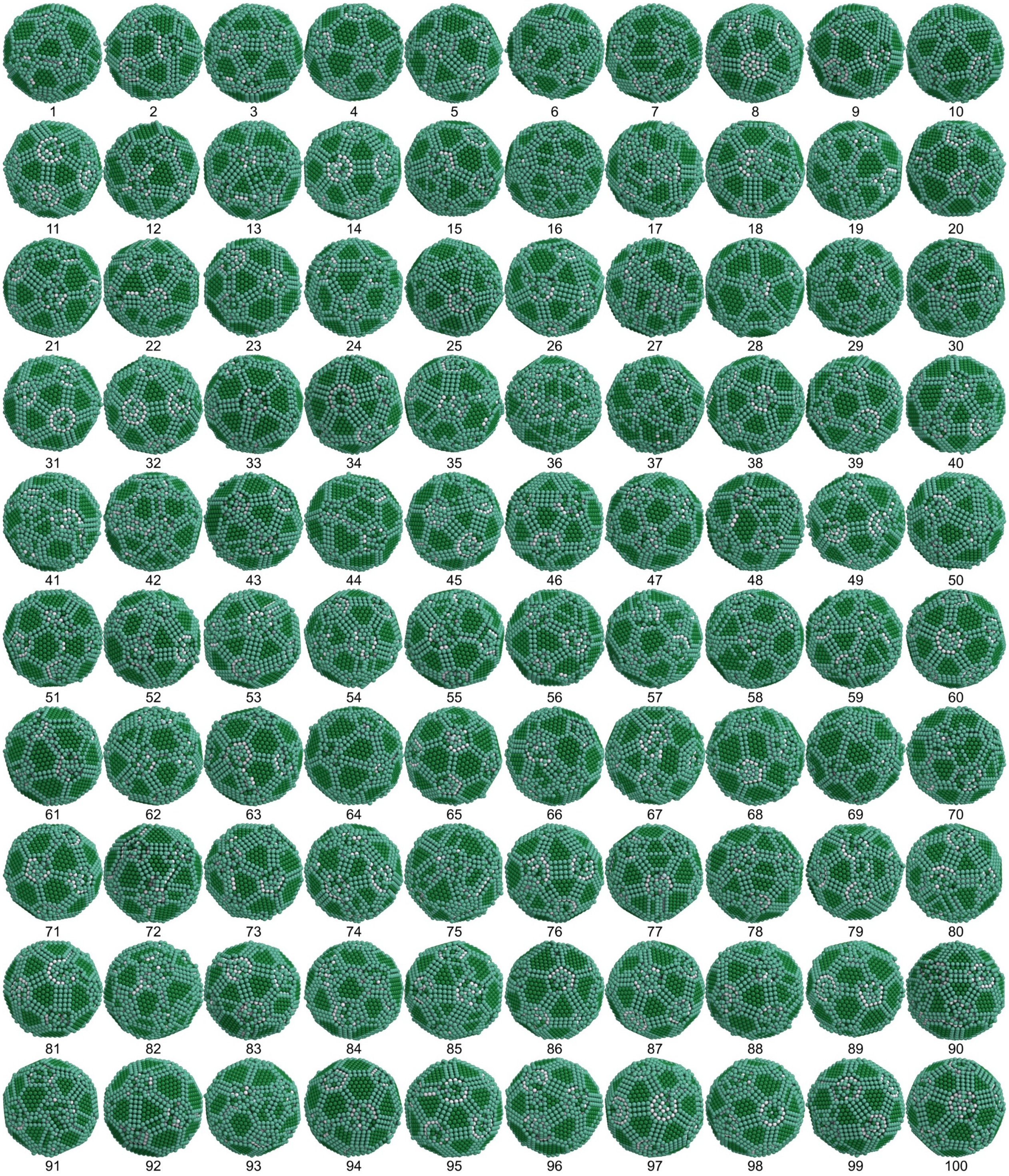

Supplementary Figure 11 | Quenched snapshots of clusters from the 100 equations of state in Fig. 5a at ϕ = 0.55, *N* = 5971 (*m* + *a* = 12 shells). Almost all clusters show characteristic surface features (rectangles and truncated triangles) and are well described as MCCs in the outer shell. Confined self-assembly into MCCs is reproducible for this system size.

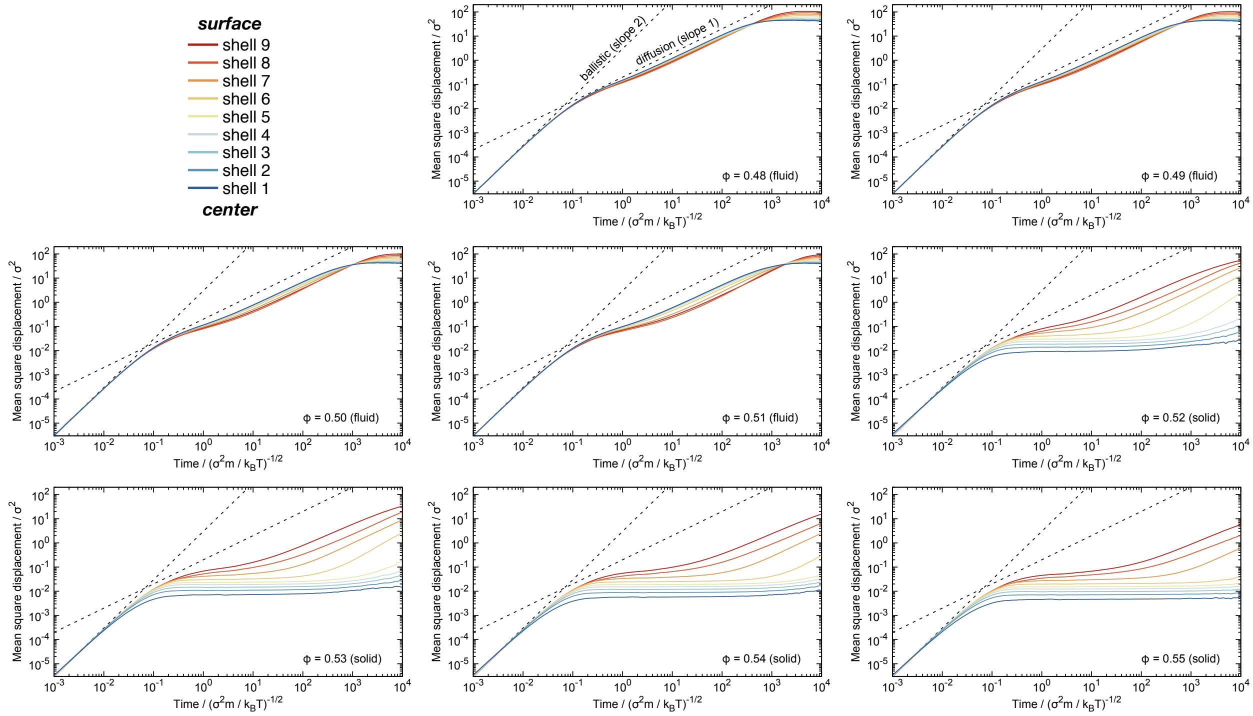

Supplementary Figure 12 | Mean square displacement of particles in different shells (counted from the center) at different packing fractions for N = 2585. The shell number corresponds to the shell the particle was part of at the start of the mean square displacement measurement. The graph at ϕ = 0.51 is shown in Fig. 5g. We can distinguish ballistic motion (slope 2), diffusive motion (slope 1), and a plateau when diffusion length reaches the size of the confinement. In the fluid phase, our simulation typically reaches the plateau over the total duration of the simulation. Diffusion is faster in inner shells. In the icosahedral cluster (solid phase), diffusion is much slower in central shells and slightly slower in shells near the surface.

1.6 Thermodynamic Stability of MCCs

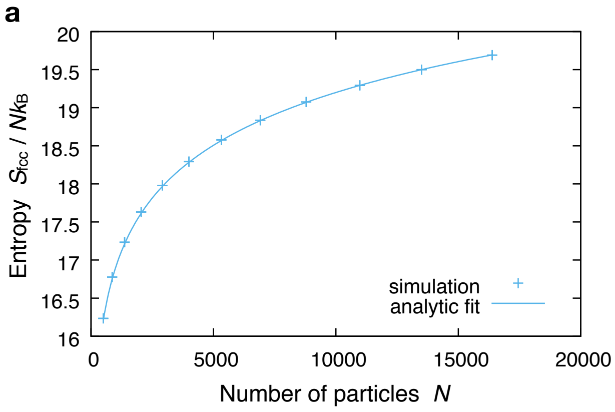

Supplementary Figure 13 | Entropy of the face-centered cubic (FCC) crystal. Entropy S is calculated with the Einstein crystal method (*14*) using periodic boundary conditions **a**, for various values of particle number N and fixed $\phi = 0.52$ and **b**, for various values of packing fraction ϕ and fixed $N = 2048$. The data is fitted for $\phi = 0.52$ (**a**) with an analytical Ansatz $S_{\mathrm{FCC}}(N, \phi)$ discussed in Simulation Methods above and also works well for $N = 2048$ up to high packing fractions (**b**). We use the information from this data to subtract the free energy bulk contribution.

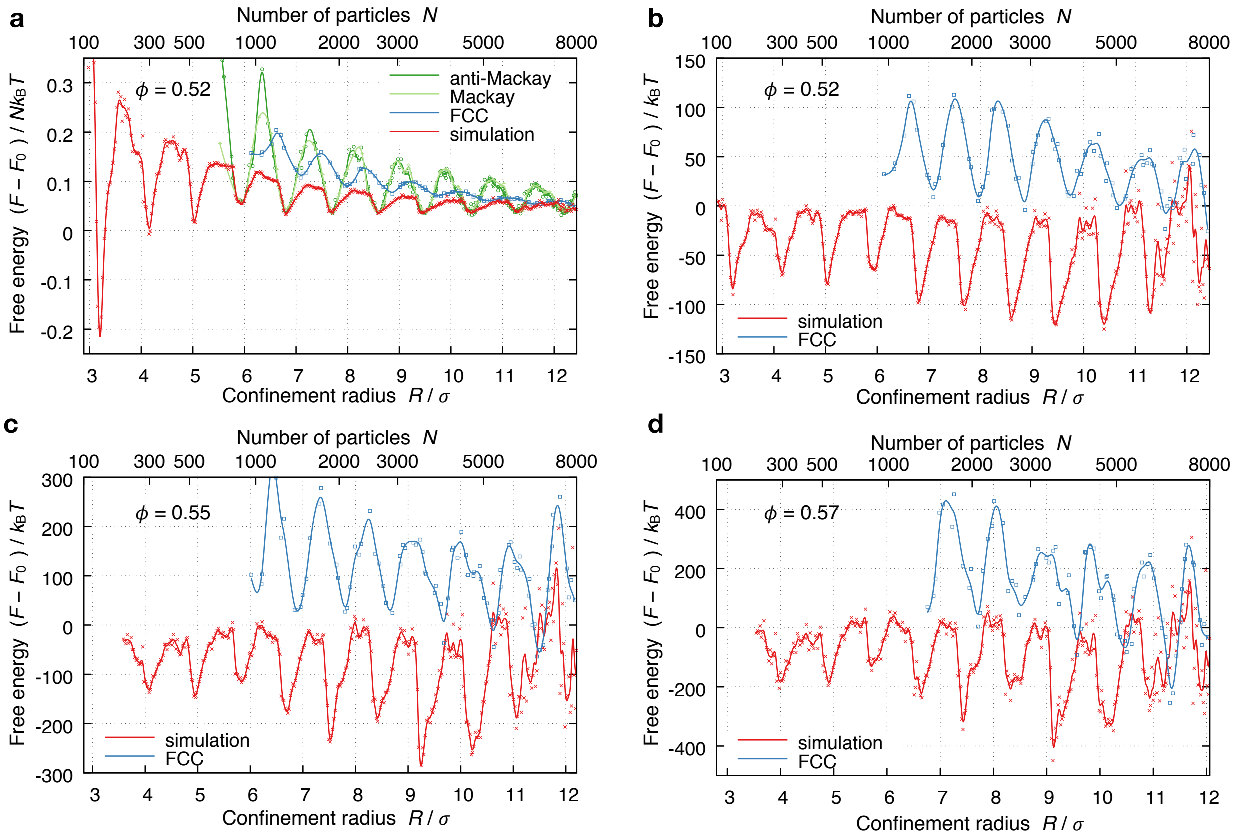

e

Shell number	3	4	5	6	7	8	9	10	11	12
Particle number N	137	298	530	881	1308	1905	2641	3530	4676	5971
Relative well depth / Nk_BT	0.6	0.22	0.15	0.07	0.065	0.05	0.04	0.033	0.026	0.018
Absolute well depth / k_BT	85	65	80	60	90	95	105	115	120	105

Supplementary Figure 14 | Thermodynamic stability of MCCs. a, Free energy of colloidal clusters computed at $\phi = 0.52$ for cluster sizes in the range $100 < N < 8000$. The free energy values are normalized by subtraction of the free energy of the bulk corrected for surface effects F_0. This figure is mostly identical to Fig. 5h but here we also include free energy values for (anti-)Mackay clusters from ideal geometric model with varying the radius of the truncation sphere (blue and green line). We observe that the curves for (anti-)Mackay clusters from the model and simulation agree in the free energy minima. Outside of the free energy minima, the clusters self-assembled in simulation have lower free energy than those constructed according to the model. Here, defect formation is crucial (see discussion in main text). **b-d**, Total free energy of the cluster, obtained by multiplying the data in Fig. 5h by the system size N at $\phi = 0.52$ (**b**), $\phi = 0.55$ (**c**), and $\phi = 0.57$ (**d**). Free energy minima deepen with increasing ϕ demonstrating that shell formation is more pronounced at higher density. Note that the parameter for the bulk contribution has been chosen differently in (**a**) and (**b**) for best visualization. This explains the different decay of the free energy as N increases. **e**, The table lists the position of the free energy minima (particle number), free energy cost per particle (relative well depth) and the total free energy gain for forming a complete shell (absolute well depth) at $\phi = 0.52$.

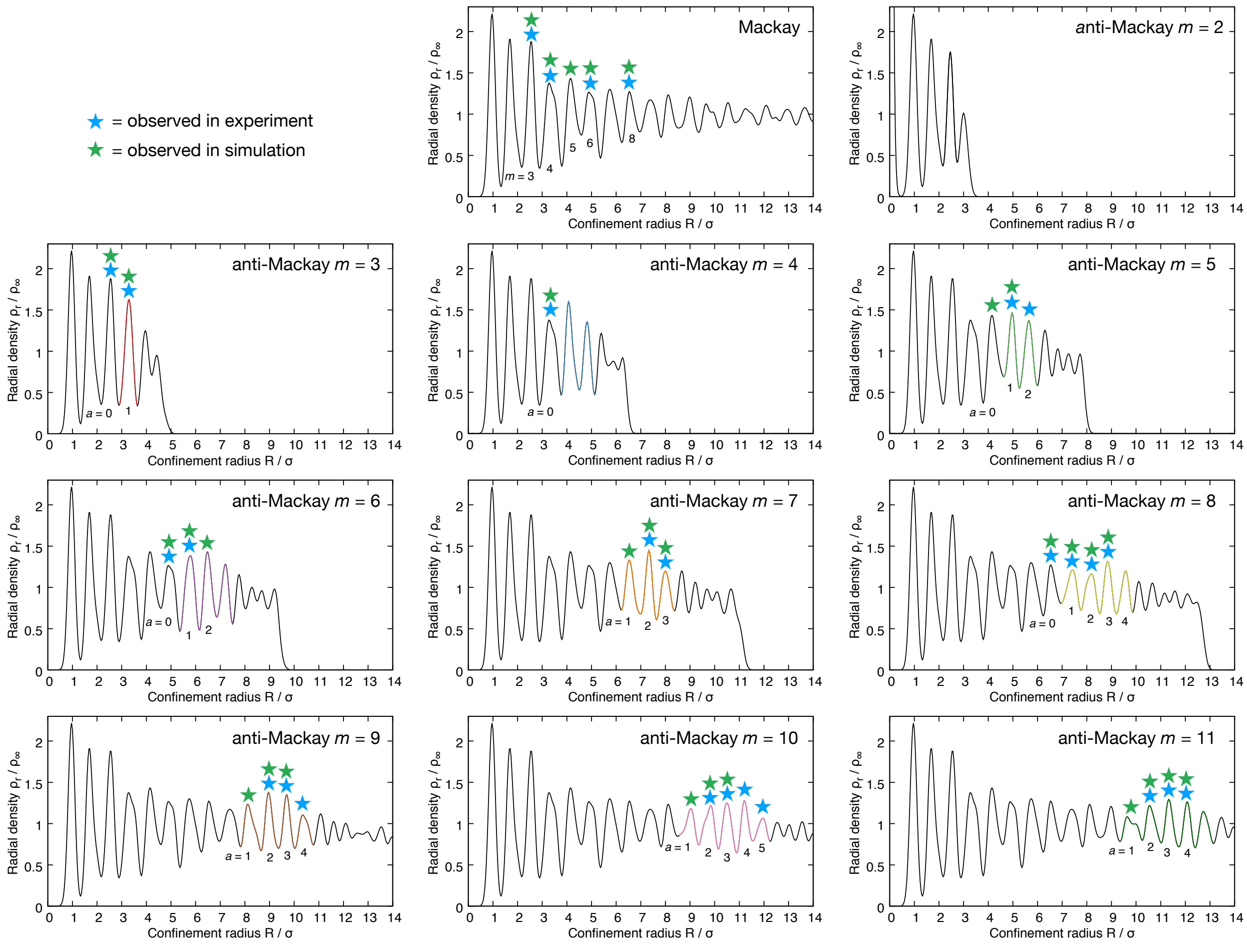

Supplementary Figure 15 | Radial density of Mackay and anti-Mackay clusters. Radial density of geometrically constructed clusters is measured from the cluster center, applying a Gaussian blur (convolution) to mimic thermal motion. A cut-off is applied when the number of anti-Mackay shells reaches the number of Mackay shells, $a = m$. Regions with $0 \leq a \leq m / 2$ are highlighted with color. Cluster types found in experiment or simulation are marked with stars. We generally observe that peaks corresponding to shells in Mackay clusters decay and smear out towards higher m. In contrast, peaks corresponding to clusters with a few anti-Mackay shells appear higher and narrower, indicating highly spherical shells. This demonstrates that while a low number of anti-Mackay shells can be preferable (they are very spherical), adding too many anti-Mackay shells typically does not bring an improvement.

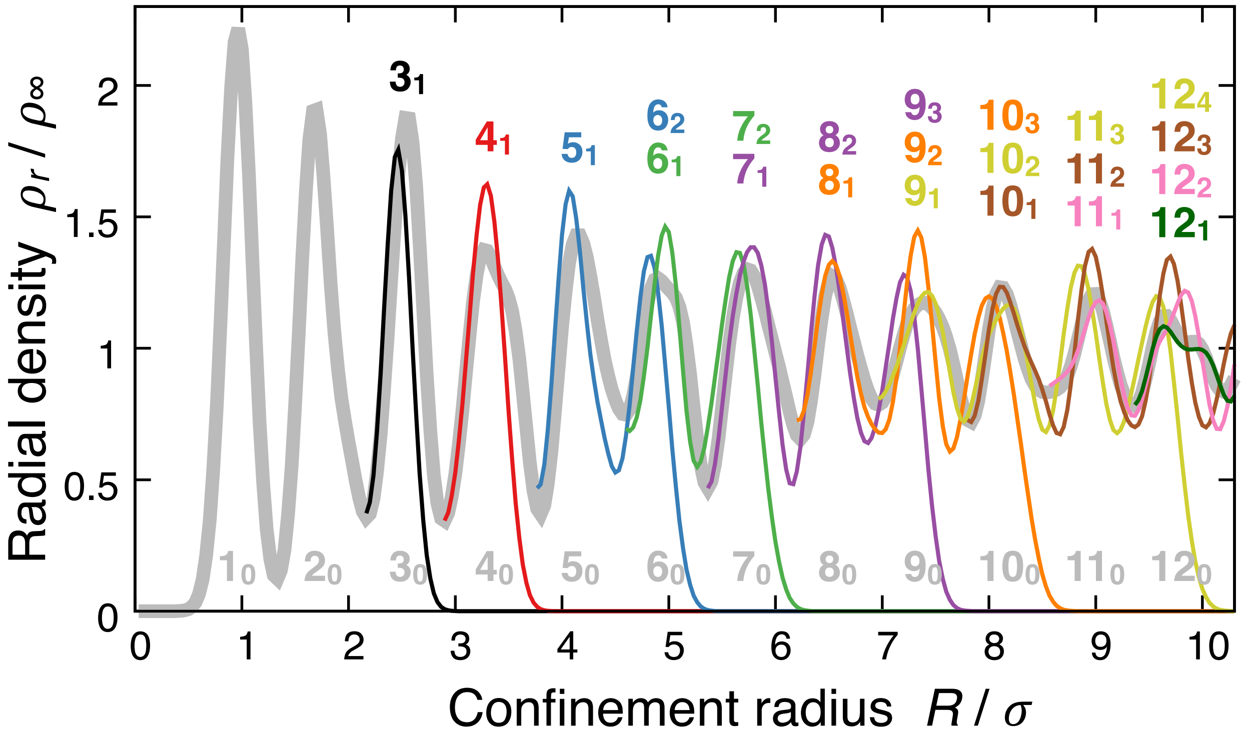

Supplementary Figure 16 | Overlay of radial density functions. The radial densities of Supplementary Figure 15 are overlaid in this figure. The radial density of Mackay cluster ($a = 0$) is shown with a thick gray line. We observe higher, narrower peaks that are often shifted to a smaller distance R for clusters with anti-Mackay shells compared to clusters without such shells. This suggests that anti-Mackay shells tend to be more compact compared to the Mackay shells, which is an important stability factor favoring the formation of a low number of anti-Mackay shells.

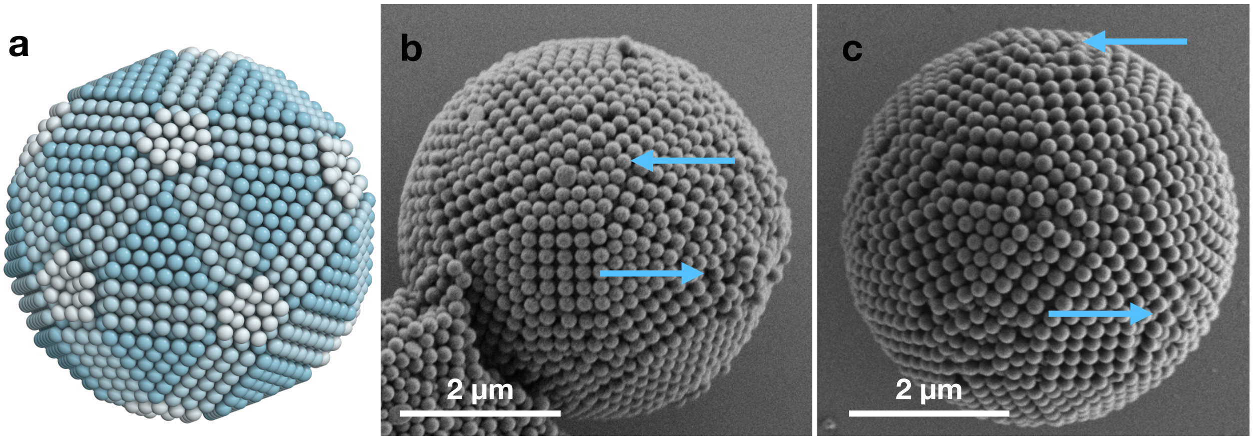

Supplementary Figure 17 | Disorder in icosahedral vertex regions. Defects tend to appear in icosahedral vertex regions in MCCs, as shown by the blue arrows in SEM images. This is predicted by the model as tetrahedra over vertex regions are more deformed than all the other parts of the model.
