# Supplementary material for: Magic number colloidal clusters as minimum free energy structures
Source: Nat Commun. 2018 Dec 10;9:5259. doi: 10.1038/s41467-018-07600-4 (PMC6288123; doi:10.1038/s41467-018-07600-4)
Supplement: Supplementary file 3 — Description of Additional Supplementary Files [file 41467_2018_7600_MOESM3_ESM.pdf]

Description of Additional Supplementary Files

Supplementary Movie 1. "BF-STEM_Tilt-Series". Bright field (BF) STEM tilt series raw data without further alignment (tilt angle range from −76° to 62° with 1° tilt increment) of a 7_2 type MCC as deposited on a Lacey carbon TEM grid. The movie shows the transition from three-fold to five-fold, two-fold, five-fold and three-fold symmetric axis as the MCC rotates, in agreement with the MCC model.

Supplementary Movie 2. "HAADF-STEM_Tilt-Series". High-angle annular dark field (HAADF) STEM tilt series after alignment (tilt angle range from −76° to 62° with 1° tilt increment) of a 72 type MCC as deposited on a Lacey carbon TEM grid. The movie shows the transition from three-fold to five-fold, two-fold, five-fold and three-fold symmetric axis as the MCC rotates, in agreement with the MCC model

Supplementary Movie 3. "3D-Reconstruction". Virtual slices through the tomographic reconstruction of the 7_2 type MCC perpendicular to its two-fold axis confirm the accuracy of the MCC model. At the beginning of the movie, the slices first show the rectangle region at the surface. As the viewing plane moves towards the center, two complete five-shell tetrahedra in mirror symmetry, two anti-Mackay shells, as well as some pentagonal patterns inherent of icosahedral nature are revealed. A detailed analysis comparing individual colloids identifies 602 out of 643 (94% accuracy) particles at their predicted position by the model in the upper hemisphere.
